# Polymorphisms in Tumour Necrosis Factor Alpha (TNF*α*) Gene in Patients with Acute Pancreatitis

**DOI:** 10.1155/2010/482950

**Published:** 2010-04-14

**Authors:** Gül Özhan, Hakan T. Yanar, Cemalettin Ertekin, Buket Alpertunga

**Affiliations:** ^1^Department of Pharmaceutical Toxicology, Faculty of Pharmacy, Istanbul University, 34116 Istanbul, Turkey; ^2^Department of General Surgery, Faculty of Medicine, Istanbul University, 34390 Istanbul, Turkey

## Abstract

Proinflammatory cytokines, such as tumour necrosis factor *α* (TNF*α*), play fundamental roles in the pathogenesis of acute pancreatitis (AP). The aim of this study was to determine if polymorphisms in the TNF*α* gene are associated with AP. Two polymorphisms located in the promoter region (positions −308 and −238) in TNF*α* gene were determined using polymerase chain reaction- (PCR-) restriction fragment length polymorphism (RFLP) methods in 103 patients with AP and 92 healthy controls. Odds ratios (ORs) and 95% confidence intervals (CI) were estimated using logistic regression analysis adjusted for age, sex, BMI and smoking. The frequencies of TNF*α* polymorphisms were both similar in patients with mild or severe pancreatitis, so were in pancreatitis patients and in controls. We suggest that both SNPs of TNF*α* are not genetic risk factor for AP susceptibility (OR = 1.63; 95% CI: 1.13−4.01 for TNF*α* ^−308^ and OR = 0.86; 95% CI: 0.75−1.77 for TNF*α* ^−238^).

## 1. Introduction

AP is a common disease whose severity varies widely from mild forms only affecting the pancreas (80% of the patients) to severe forms associated with multiple organ failure (20% of the patients). The reported incidence is approximately 30–40 per 100 000 population per year and 25% will develop severe or life threatening complications [1, 2]. The mechanisms responsible for the development of pancreatitis have not yet been fully elucidated. However, it has been clearly known that one of the important factors, activating pancreatic stellate cells during pancreatic injury, is proinflammatory cytokines known to be upregulated early in the course of acute pancreatic inflammation [3, 4]. Genetic factors, especially related to cytokines, may play important roles in susceptibility to pancreatic injury, as well as in the severity and evolution of the inflammatory process [5]. 

Tumour necrosis factor *α* (TNF*α*), thought to be the first cytokine released, is a principal mediator of immune responses [6]. The gene that codifies for TNF*α* is located within the major histocompatibility complex (MHC) class III region in the short arm of the human sixth chromosome [7]. Two biallelic polymorphisms in the promoter region of the TNF*α* gene may be relevant for the production of TNF [8]. The first one at the position −238 nucleotides relative to the transcriptional start site lies within a putative regulator sequence where a G to A substitution defines two variants (TNF*α* ^−238^ G and TNF*α* ^−238^ A). The second polymorphism at position −308 is also a point mutation, where the presence of G defines the common variant TNF*α* ^−308^ G and A defines the less common variant TNF*α* ^−308^ A [9]. Although some researches have showen that SNPs of TNF*α* are associated with inflammatory diseases in pancreas, identification of an association between the polymorphisms of TNF*α* and susceptibility to AP is less clear [10–13]. In the present study, our aim was to determine the association between TNF*α* promoter polymorphism (TNF*α* ^−308^; TNF*α* ^−238^) and AP susceptibility in patients with AP and controls nested within the Hospital of Istanbul University, Turkey. We hypothesized these polymorphisms could influence the risk of AP. This article can be significant for the approach to the possible relationship between key inflammatory gene polymorphisms and pancreatitis.

## 2. Material and Methods

### 2.1. Study Population

Blood samples were collected from 103 patients with AP seen at the Emergency Service, the Hospital of Istanbul University, Turkey between March 2008 and March 2009. The criteria for diagnosis of AP were: a clinical history consistent with the disease, appropriate radiological evidence, and serum amylase level greater than 3 times the upper limit of normal. The progress of individuals with regard to development of complications was monitored during their disease episode. The disease severity was classified as mild or severe according to criteria defined by the Atlanta Consensus Conference [14]. Cases consisted of 68 patients with mild and 35 patients with severe AP. As a control group, 92 healthy ethnically matched subjects were obtained during the same period to examine association between TNF*α* genotypes and susceptibility to pancreatitis. The controls showed no evidence of pancreatitis or cancer. Ethical approval for the study was obtained from the Local Research Ethics Committee, Istanbul, Turkey. All participants provided written informed consent. We recorded the smoking status in addition to age, BMI, sex and family history. For smoking status, a person who had smoked at least once a day for >1 year in his or her lifetime was regarded as a smoker. 

### 2.2. Selection of Single Nucleotide Polymorphisms

The human TNF*α* gene situated on the short arm of chromosome 6 at p21.3 locus [7]. We collected data on TNF*α* polymorphisms from publicly available databases, such as dbSNP (http://www.ncbi.nlm.nih.gov/) and HapMap Phase II database (http://hapmap.org/index.html.en). The studied SNPs (TNF*α* ^−308^ (rs1800629) and TNF*α* ^−238^ (rs361525)) were chosen according to the following criteria: (a) minor allele frequency of 5% or greater in Caucasians, according to literature data, (b) previous epidemiologic findings indicating associations with pancreatitis and cancer susceptibility.

### 2.3. Genotyping

Genomic DNA was extracted from blood samples using a sodium perchlorate/chloroform extraction as described by Daly et al. [15]. Genotypes of TNF*α* polymorphisms were determined by PCR-RFLP methods. Genotyping was performed without knowledge of subjects' patients or control status. The sequences of primers were designed by GenBank (accession numbers; NT007592; NM000594). For PCR amplification, genomic DNA (100 ng–1 *μ*g) was added to 25 *μ*L of PCR master-mix as described by Daly et al. [15]. The PCR products were digested with the various restriction enzymes. Digestion products were analysed by electrophoresis on 10% polyacrylamide gels with TBE buffer. Band patterns representing the various genotypes are summarized in below.

For TNF*α* ^−308^ G > A (rs1800629), primers were 5′-ATC TGG AGG AAG CGG TAG TG-3′ and 5′-AAT AGG TTT TGA GGG CCA TG-3′. The following PCR protocol was used: 94°C for 3 minutes; 35 cycles of 94°C for 30 seconds, 55°C for 30 seconds, 72°C for 30 seconds; 72°C for 5 minutes. The PCR product was directly digested with 2 U *NcoI* restriction enzyme at 37°C for overnight. Digestion with *Nco*I produced an uncut 222-bp fragment from the mutant allele (TNF*α* ^−308^ A), 130- and 20-bp fragments from the wild-type allele (TNF*α* ^−308^ G). 

For TNF*α* ^−238^ G > A (rs361525), primers were 5′-ATC TGG AGG AAG CGG TAG TG-3′ and 5′-AGA AGA CCC CCC TCG GAA CC-3′. The following PCR protocol was used: 94°C for 3 minutes; 35 cycles of 94°C for 30 seconds, 57°C for 30 seconds, 72°C for 30 seconds; 72°C for 5 minutes. The PCR product was directly digested with 2 U *Msp*I restriction enzyme at 37°C for overnight. Digestion with *Msp*I produced an uncut 150-bp fragment from the mutant allele (TNF*α* ^−238^ A), 130- and 20-bp fragments from the wild-type allele (TNF*α* ^−238^ G). 

To assess reliability of genotyping, analysis repeated on 10% of the samples.

### 2.4. Statistical Analysis

*S*tatistical analysis was conducted by using the computer software *Statistical Package for Social Sciences-*SPSS for windows (version 13.0) (Chicago, IL). ORs and 95% CIs were estimated by conditional logistic regression analyses based on the comparison of genotypes between patients with AP and healthy controls, adjusting for the potential confounders, age, BMI, sex, smoking. Hardy-Weinberg equilibrium was tested to compare the observed and expected genotype frequencies among cases and controls, respectively. A two-sided *P*-value <.05 was considered to be statistically significant. For analyses of genotype frequencies, the wild-type category (chosen either as the most common wild-type frequency or arbitrarily if both alleles showed similar frequencies) was the reference group. 

## 3. Results

There were no significant differences between the cases and controls for the mean age and sex distribution, and this suggested that the matching based on these two variables was adequate. The mean age (±SD) was 52.7 (±15.1) years for patients and 46.7 (±16.4) years for controls (*P* = .729). In this study, a significant association was observed for interaction between BMI and AP risk (OR = 3.07; 95% CI: 1.68−5.60; *P* = .0002) (Table 1). As it can be seen in Table 2, no association between TNF*α* genotypes and family history of pancreatitis or any cancer was detected in our study. Firstly, we tested the association between two TNF*α* SNPs (TNF*α* ^−308^, TNF*α* ^−238^) and AP risk in our study of 103 cases and 92 controls. TNF*α* ^−308^ A and TNF*α* ^−238^G, the highest allele frequencies observed in our study, were 0.616 and 0.870 in cases compared with 0.587 and 0.891 in controls, respectively. The observed genotype frequencies of all seven SNPs in cases and controls conformed to the Hardy-Weinberg equilibrium. We then analyzed the differences between cases and controls in the distribution of genotype. Genotype distribution of two SNPs (rs1800629; rs361525) in cases and controls did not significantly differ (*P* > .05) and thus these polymorphisms were not associated with risk of pancreatic cancer (Table 2). Similarly, there was no significant difference in the distribution of two TNF*α* gene polymorphisms studied between patients with mild or severe diseases. Finally, we investigated the associations between smoking status both AP risk and the two TNF*α* polymorphisms. Smoking was slightly associated with the risk of AP (OR = 1.70; 95% CI: 0.95−3.04; *P* = .075) while it was no observation any association between smoking and the studied polymorphisms (*P* = .1). 

## 4. Discussion

The link between inflammation and the development of cancer has been recognized by a number of researchers since Rudolf Virchow noted leukocytes in neoplastic tissue and suggested that there was a connection between cancer and inflammation in 1863 [16, 17]. Although many forms of pancreatitis have been linked to a higher risk for pancreatic cancer, the relationship between the two is ill-defined [18, 19]. The course of chronic pancreatitis is characterized by recurrent episodes of AP, which cause parenchymal injury and necrosis, with increasing amounts of fibrosis, chronic inflammation, and parenchymal cell loss with each successive episode [20, 21]. Patients with chronic pancreatitis have an increased risk of developing pancreatic cancer with a rate 15- to 16-fold greater than that of the general population [20]. It can be seen that AP is first step in this pathway progressed from inflammation to cancer in pancreas. However, inflammation alone usually does not lead to cancer. Several factors have been implicated for the recent rise in the frequency of AP, including age, alcohol consumption, smoking, gallstones, diet high in animal fat, and genetic disorders [21, 22]. On the contrary to the results obtained from some researches [23, 24]. No association between the risk of AP and family history was detected in this study (Table 1). It is indicated that smoking status, duration and intensity were risk factors for AP by the researchers [25–27]. We found that there was a weak association between cigarette smoking and development of AP in the study population (OR = 1.59; 95% CI: 0.89−2.84; *P* = .075) (Table 1). Further investigation with more samples would be better to determine strictly this association. As to BMI, a significant association was observed for interaction between BMI and AP risk (OR = 3.07; 95% CI: 1.68−5.60; *P* = .0002) similar to the results obtained from Lindkvist et al. [28] and Gumbs [29]. However, there was no specifically interaction between any studied polymorphisms and BMI. In two SNPs, the risk of AP was higher than <2-fold from the point of view of BMI (no data shown). The main limitation of our study is the relative small study size, which may result in less precise estimation of gene-environment interaction in AP. 

TNF*α* expression in the pancreas is increased by the onset of experimental pancreatitis, and antagonism of TNF*α* reduces the severity of local pancreatic inflammation. Levels of soluble TNF*α* receptors, indicators of TNF*α* activity, have been found to be increased in patients with severe disease, and TNF*α* blockade has been shown to reduce mortality and ameliorate markers of severe systemic disease in experimental AP [30–34]. TNF*α* polymorphisms have been associated with susceptibility and outcome of various infectious, inflammatory and neoplastic diseases [35–37]. As to AP, identification of an association between the polymorphisms of TNF*α* and susceptibility to AP is less clear, although several SNPs in TNF*α* have been reported previously in both many forms of pancreatitis and pancreatic cancer. A significant association between TNF*α* at position −308 and −238 has recently been described in patients with chronic pancreatitis and with pancreatic cancer [13, 14, 38, 39] while some researches have reported no association between TNF*α* polymorphism and susceptibility chronic pancreatitis or pancreatic cancer [40–42]. Beranek et al. [43] found that TNF*α* ^−238^ might be a relevant risk factor for disease manifestation in families with hereditary pancreatitis. On the contrary, there was no association between TNF*α* ^−238^ polymorphism and the risk of AP (OR = 0.86; 95% CI: 0.75−1.77; *P* = .175) in the study (Table 2). Balog et al. [12] obtained that TNF*α* ^−308^ A alleles were associated with risk of AP. They noticed that genotype assessments may be important prognostic tools to predict disease severity and the course of AP. However, Zhang et al. [44] found no association between AP and TNF*α* ^−308^ in Chinese population with AP. But, they suggest that TNF*α* ^−308^ A allele is associated with a susceptibility to severe sepsis complicating of AP. TNF*α* ^−308^was genotyped in AP patients in UK population by Sargen et al. [45] and Powell et al. [46]. They found that TNF*α* ^−308^ plays no part in determination of disease severity or susceptibility to AP. Similarly, we found there was no association between TNF*α* ^−308^ polymorphism and the risk of AP (OR = 1.63; 95% CI: 1.13−4.01; *P* = .145) (Table 2). We have also observed that the TNF*α* gene polymorphisms studied were not associated with disease severity in AP (no data shown). 

In conclusion, we obtained the results on the contrary to our hypothesis. It is unlikely that the studied SNPs (TNF*α* ^−308^ and TNF*α* ^−238^) will regulate AP. In complex biologic disease processes such as AP, the effect of an SNP in inflammation process may be minimized through the interaction of other factors. Also, there are several potential reasons for failing to observe any association between TNF*α* gene polymorphisms studied and levels of disease severity. It is, perhaps, therefore not surprising that single gene polymorphisms do not correlate with outcome in patients with mild or severe AP. Therefore, additional studies on a larger group of patients will be required to confirm these findings. Besides, further studies on the level of expression of TNF*α* gene and on the other potential SNPs in neighbouring genes and in other inflammatory cytokines may likely provide additional insight into the role and implications of alterations in individual susceptibility to AP. We believe that any clarification of mechanisms linking inflammation and cancers will be beneficial to the development of efficacious prevention and therapies of inflammation-associated cancers.

## Figures and Tables

**Table 1:** Distribution of select characteristics among AP patients and controls.

	Cases (%) *n* = 103	Control (%) *n* = 92	OR (95% CI)	*P*-value
Gender				
Male	55 (53.4)	45 (48.9)	1.00 (reference)	.532
Female	48 (46.6)	47 (51.1)	1.2 (0.68−2.10)	
Age				
≤60	30 (29.1)	25 (27.2)	1.00 (reference)	.729
>60	72 (69.9)	67 (72.8)	0.90 (0.48−1.68)	
Missing	1 (1.0)	*—*		
Family history				
No	80 (77.7)	82 (89.1)	1.00 (reference)	.150
Yes	18 (17.5)	10 (10.9)	1.85 (0.80−4.24)	
Missing	5 (4.9)			
BMI*				
≤25	48 (46.6)	25 (27.2)	1.00 (reference)	.0002
>25	55 (53.4)	67 (72.8)	3.07 (1.68−5.60)	
Smoking status				
Non-smoking	57 (55.3)	63 (68.5)	1.00 (reference)	
Smoking	46 (44.7)	30 (32.6)	1.70 (0.95−3.04)	.075

*BMI; body mass index (kg/m^2^).

**Table 2:** Genotype frequencies of TNF*α* among cases and controls and the association with AP risk. ORs with 95% CI and *P*-values were calculated for wild/wild genotype versus wild/mutant and mutant/mutant genotypes.

TNF*α* polymorphisms	Genotype	Cases (%) *n* = 103	Controls (%) *n* = 92	OR (95% CI)*	*P*-value
TNF*α* ^−308^G > A rs1800629	GG	15 (14.6)	21 (22.8)	GG versus any A	.145
	GA	43 (41.8)	34 (37.0)	1.63 (1.13−4.01)	
	AA	45 (43.7)	37 (40.2)		

TNF*α* ^−238^G > A rs361525	GG	90 (87.4)	73 (79.4)	GG versus any A	.175
	GA	8 (7.8)	18 (19.6)	0.86 (0.75−1.77)	
	AA	5 (4.9)	1 (1.1)		

*OR: odds ratio; CI: 95% confidence interval; OR adjusted for age, sex, BMI, smoking status for logistic regression analysis.

## References

[1] Forsmark CE, Toskes PP (1995). Acute pancreatitis: medical management. *Critical Care Clinics*.

[2] McKay CJ, Evans S, Sinclair M, Carter CR, Imrie CW (1999). High early mortality rate from acute pancreatitis in Scotland, 1984–1995. *British Journal of Surgery*.

[3] Norman J (1998). Role of cytokines in the pathogenesis of acute pancreatitis. *American Journal of Surgery*.

[4] Formela LJ, Galloway SW, Kingsnorth AN (1995). Inflammatory mediators in acute pancreatitis. *British Journal of Surgery*.

[5] Weiss FU, Simon P, Mayerle J, Kraft M, Lerch MM (2006). Germline mutations and gene polymorphism associated with human pancreatitis. *Endocrinology and Metabolism Clinics of North America*.

[6] Grewal HP, Kotb M, El Din AM (1994). Induction of tumor necrosis factor in severe acute pancreatitis and its subsequent reduction after hepatic passage. *Surgery*.

[7] Spies T, Morton C, Nedospasov SA (1986). Genes for tumour necrosis factor alpha and beta are linked to the major histocompatibility complex. *Proceedings of the National Academy of Sciences of the United States of America*.

[8] D’Alfonso S, Richiardi PM (1994). A polymorphic variation in a putative regulation box of the TNFA promoter region. *Immunogenetics*.

[9] Wilson AG, di Giovane FS, Blakemore AIF (1992). Single base polymorphism in the human necrosis alpha (TNF-*α*) gene detectable by NcoI restriction of PCR product. *Human Molecular Genetics*.

[10] Duell EJ, Casella DP, Burk RD, Kelsey KT, Holly EA (2006). Inflammation, genetic polymorphisms in proinflammatory genes TNF-A, RANTES, and CCR5, and risk of pancreatic adenocarcinoma. *Cancer Epidemiology Biomarkers and Prevention*.

[11] O’Reilly DA, Dunlop S, Sargen K, Demaine A, Wilkinson S, Kingsnorth AN (2006). Tumour necrosis factor microsatellite haplotypes are associated with chronic pancreatitis. *Journal of the Pancreas*.

[12] Balog A, Gyulai Z, Boros LG (2005). Polymorphism of the TNF-alpha, HSP70-2, and CD14 genes increases susceptibility to severe acute pancreatitis. *Pancreas*.

[13] Dianliang Z, Jieshou L, Zhiwei J, Baojun Y (2003). Association of plasma levels of tumor necrosis factor (TNF)-*α* and its soluble receptors, two polymorphisms of the TNF gene, with acute severe pancreatitis and early septic shock due to it. *Pancreas*.

[14] Bradley EL, Frey CF (1993). A clinically based classification system for acute pancreatitis. *Archives of Surgery*.

[15] Daly AK, Steen VM, Fairbrother KS, Idle JR (1996). CYP2D6 multiallelism. *Methods in Enzymology*.

[16] Balkwill F, Mantovani A (2001). Inflammation and cancer: back to Virchow?. *The Lancet*.

[17] Lu H, Ouyang W, Huang C (2006). Inflammation, a key event in cancer development. *Molecular Cancer Research*.

[18] Jura N, Archer H, Bar-Sagi D (2005). Chronic pancreatitis, pancreatic adenocarcinoma and the black box in-between. *Cell Research*.

[19] Lowenfels AB, Maisonneuvi P, Cavallini G (1993). Pancreatitis and the risk of pancreatic cancer. *The New England Journal of Medicine*.

[20] Omary MB, Lugea A, Lowe AW, Pandol SJ (2007). The pancreatic stellate cell: a star on the rise in pancreatic diseases. *Journal of Clinical Investigation*.

[21] Zabel-Langhennig A, Holler B, Engeland K, Mössner J (1999). Cyclooxygenase-2 transcription is stimulated and amylase secretion is inhibited in pancreatic acinar cells after induction of acute pancreatitis. *Biochemical and Biophysical Research Communications*.

[22] Garcea G, Dennison AR, Steward WP, Berry DP (2005). Role of inflammation in pancreatic carcinogenesis and the implications for future therapy. *Pancreatology*.

[23] Heinig J, Simon P, Baas S, Lerch MM (2002). Hereditary pancreatitis—a rare differential diagnosis in patients with menstruation-associated recurrent acute pancreatitis: a case report. *Gynecological Endocrinology*.

[24] Rebours V, Boutron-Ruault MC, Jooste V, Bouvier AM (2008). Mortality rate in patients with hereditary pancreatitis (HP),compared with the french general population. *Gastroenterology*.

[25] Tolstrup JS, Kristiansen L, Becker U, Gronbaek M (2009). Smoking and risk of acute and chronic pancreatitis among women and men. *Archives of Internal Medicine*.

[26] Yadav D, Hawes RH, Brand RE (2009). Alcohol consumption, cigarette smoking, and the risk of recurrent acute and chronic pancreatitis. *Archives of Internal Medicine*.

[27] Lochan R, Daly AK, Reeves HL, Charnley RM (2008). Genetic susceptibility in pancreatic ductal adenocarcinoma. *British Journal of Surgery*.

[28] Lindkvist B, Appelros S, Manjer J (2008). A prospective cohort study of smoking in acute pancreatitis. *Pancreatology*.

[29] Gumbs AA (2008). Obesity, pancreatitis, and pancreatic cancer. *Obesity Surgery*.

[30] Norman JG, Fink GW, Franz MG (1995). Acute pancreatitis induces intrapancreatic tumor necrosis factor gene expression. *Archives of Surgery*.

[31] Fu K, Sarras MP, De Lisle RC (1997). Expression of oxidative stressresponsive genes and cytokine genes during cerulein-induced acute pancreatitis. *American Journal of Physiology*.

[32] Hughes CB, Gaber LW, Mohey el-Din AB (1996). Inhibition of TNF-*α* improves survival in an experimental model of acute pancreatitis. *American Journal of Surgery*.

[33] De Beaux AC, Goldie AS, Ross JA, Carter DC, Fearon KCH (1996). Serum concentrations of inflammatory mediators related to organ failure in patients with acute pancreatitis. *British Journal of Surgery*.

[34] Norman JG, Fink GW, Messina J, Carter G, Franz MG (1996). Timing of tumor necrosis factor antagonism is critical in determining outcome in murine lethal acute pancreatitis. *Surgery*.

[35] Mira J-P, Cariou A, Grall F (1999). Association of TNF2, a TNF-*α* promoter polymorphism, with septic shock susceptibility and mortality: a multicenter study. *Journal of the American Medical Association*.

[36] Mulcahy B, Waldron-Lynch F, McDermott MF (1996). Genetic variability in the tumor necrosis factor-lymphotoxin region influences susceptibility to rheumatoid arthritis. *American Journal of Human Genetics*.

[37] Chouchane L, Ahmed SB, Baccouche S, Remadi S (1997). Polymorphism in the tumor necrosis factor-*α* promotor region and in the heat shock protein 70 genes associated with malignant tumors. *Cancer*.

[38] Witt H, Apte MV, Keim V, Wilson JS (2007). Chronic pancreatitis: challenges and advances in pathogenesis, genetics, diagnosis, and therapy. *Gastroenterology*.

[39] Chang Y-T, Chang M-C, Su T-C (2008). Association of Cystic Fibrosis Transmembrane Conductance Regulator (CFTR) mutation/variant/haplotype and Tumor Necrosis Factor (TNF) promoter polymorphism in hyperlipidemic pancreatitis. *Clinical Chemistry*.

[40] Farkas G, Hofner P, Balog A (2007). Relevance of transforming growth factor*α*1, interleukin-8, and tumor necrosis factor-*α* polymorphisms in patients with chronic pancreatitis. *European Cytokine Network*.

[41] Schneider A, Pogue-Geile K, Barmada MM, Myers-Fong E, Thompson BS, Whitcomb DC (2003). Hereditary, familial, and idiopathic chronic pancreatitis are not associated with polymorphisms in the tumor necrosis factor *α* (TNF-*α*) promoter region or the TNF receptor 1 (TNFR1) gene. *Genetics in Medicine*.

[42] Bendicho MT, Guedes JC, Silva NN (2005). Polymorphism of cytokine genes (TGF-beta1, IFN-gamma, IL-6, IL-10, and TNF-alpha) in patients with chronic pancreatitis. *Pancreas*.

[43] Beranek H, Teich N, Witt H (2003). Analysis of tumour necrosis factor alpha and interleukin 10 promotor variants in patients with chronic pancreatitis. *European Journal of Gastroenterology and Hepatology*.

[44] Zhang D, Li J, Jiang ZW, Yu B, Tang X (2003). Association of two polymorphisms of tumor necrosis factor gene with acute severe pancreatitis. *Journal of Surgical Research*.

[45] Sargen K, Demaine AG, Kingsnorth AN (2000). Cytokine gene polymorphisms in acute pancreatitis. *Journal of the Pancreas*.

[46] Powell JJ, Fearon KCH, Siriwardena AK, Ross JA (2001). Evidence against a role for polymorphisms at tumor necrosis factor, interleukin-1 and interleukin-1 receptor antagonist gene loci in the regulation of disease severity in acute pancreatitis. *Surgery*.

